# Comparative Analysis of Monoaxial and Polyaxial Pedicle Screws in the Surgical Correction of Adolescent Idiopathic Scoliosis

**DOI:** 10.3390/jcm13092689

**Published:** 2024-05-03

**Authors:** Jae Hyuk Yang, Hong Jin Kim, Tae Yeong Chang, Seung Woo Suh, Dong-Gune Chang

**Affiliations:** 1 Department of Orthopaedic Surgery, Korea University Anam Hospital, College of Medicine, Korea University, Seoul 02841, Republic of Korea; kuspine@korea.ac.kr (J.H.Y.); taeyeong95@naver.com (T.Y.C.); 2 Department of Orthopaedic Surgery, Inje University Sanggye Paik Hospital, College of Medicine, Inje University, Seoul 01757, Republic of Korea; hongjin0925@naver.com; 3 Department of Orthopedic Surgery, Korea University Guro Hospital, College of Medicine, Korea University, Seoul 08308, Republic of Korea; spine@korea.ac.kr

**Keywords:** adolescent idiopathic scoliosis, polyaxial pedicle screws, monoaxial pedicle screws, correction, rod derotation

## Abstract

**Background**: Although several biomechanical studies have been reported, few clinical studies have compared the efficacy of monoaxial and polyaxial pedicle screws in the surgical treatment of adolescent idiopathic scoliosis (AIS). This study aims to compare the radiological and clinical outcomes of mono- and polyaxial pedicle screws in the surgical treatment of AIS. **Methods**: A total of 46 AIS patients who underwent surgery to treat scoliosis using pedicle screw instrumentation (PSI) and rod derotation (RD) were divided into two groups according to the use of pedicle screws: the monoaxial group (*n* = 23) and polyaxial group (*n* = 23). **Results**: The correction rate of the main Cobb’s angle was higher in the monoaxial group (70.2%) than in the polyaxial group (65.3%) (*p* = 0.040). No differences in the rotational correction of the apical vertebra were evident between the two groups. SRS-22 scores showed no significant differences according to the type of pedicle screws used. **Conclusions**: The use of polyaxial pedicle screws resulted in coronal, sagittal, and rotational correction outcomes comparable to those associated with the use of monoaxial pedicle screws for surgical treatment using PSI and RD to treat moderate cases of AIS.

## 1. Introduction

Rod derotation (RD) is a commonly used maneuver in the surgical correction of adolescent idiopathic scoliosis (AIS) [1,2,3,4]. The introduction of pedicle screw instrumentation (PSI) in the thoracic spine by Suk et al. made three-dimensional correction possible due to a powerful fixation and posteromedialization effect in the surgical treatment of AIS [2,5]. The RD technique is based on a pre-bent rod’s derotation by placing the concave side of a rotated rod against the coronal scoliotic deformity to convert it into thoracic kyphosis (TK) and/or lumbar lordosis (LL) [1,6].

Biomechanical studies for pedicle screws have confirmed that their use results in superior stability and fewer implant failures compared with other instrumentation systems [7,8,9,10,11]. However, some controversies remain regarding deformity correction and the choice of pedicle screw type when treating AIS. As monoaxial pedicle screws provide greater leverage compared with polyaxial pedicle screws, RD using monoaxial pedicle screws can provide powerful correction of scoliotic curves [8]. However, monoaxial pedicle screws have proven difficult to use intraoperatively with rod engagement to pedicle screws, which may limit the ability to manipulate sagittal contours in RD procedures [10]. With the development of the instrumentation system, uniplanar pedicle screws were introduced to allow freedom of motion in the sagittal plane, which provide superior correction of the thoracic kyphosis [10,12,13,14].

The use of polyaxial pedicle screws in the surgical treatment of AIS has been viewed as controversial for a decade, since Kukulo et al. in 2005 suggested comparable efficacy between monoaxial and polyaxial pedicle screws, while Lam et al. in 2013 claimed that polyaxial pedicle screws would have less power for the derotation and reduction of the scoliotic curve [7,8]. Given the paucity of research and conflicting data, this study compares the radiological and clinical outcomes of mono- and polyaxial pedicle screws in the surgical treatment of AIS.

## 2. Materials and Methods

Under an institutional review board-approved protocol and guidance from Strengthening the Reporting of Observational Studies, this retrospectively controlled comparative study was conducted at a single institution where spinal deformity corrections are routinely performed [15].

### 2.1. Study Design, Patient Groups, and Inclusion/Exclusion Criteria

All operations were conducted by a single surgeon with vast experience in scoliosis surgery. Given the bias effect of surgical proficiency on the use of different types of pedicle screws on surgical outcomes, we enrolled AIS patients who underwent deformity correction using monoaxial pedicle screws between January 2010 and December 2010 and those who underwent deformity correction using polyaxial pedicle screws between January 2015 and December 2015.

**Figure 1:**
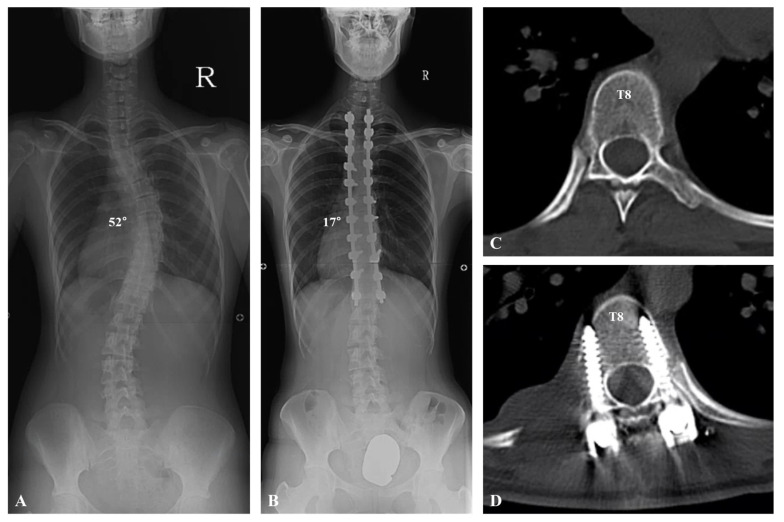
A 15-year-old female patient visited the hospital for progressive spinal deformity. (**A**) The whole-spine anteroposterior image showed 52° of scoliosis deformity with T8 apical vertebra. (**B**) Deformity correction from T2 to T12 was performed using monoaxial pedicle screws and rod derotation, producing a correction from 52° to 17°. (**C**,**D**) Axial view of computed tomography presented the apical vertebra (T8) before and after deformity correction.

**Figure 2:**
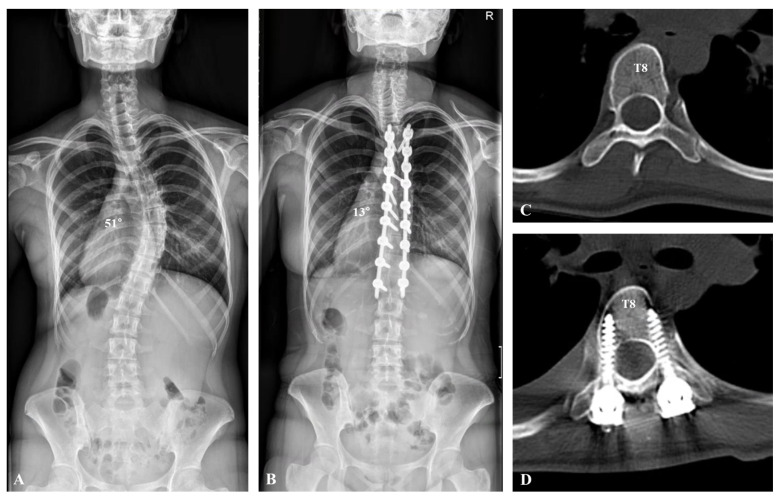
A 14-year-old female patient visited the hospital for progressive spinal deformity. (**A**) A whole-spine anteroposterior image showed 51° of scoliosis deformity with T8 apical vertebra. (**B**) Deformity correction from T5 to L1 was performed with polyaxial pedicle screws and rod derotation, resulting in a correction from 51° to 13°. (**C**,**D**) Axial view of computed tomography presented the apical vertebra (T8) before and after deformity correction.

All patients were enrolled according to the same inclusion and exclusion criteria. The inclusion criteria of this study were as follows: 11 to 17 years old, a Risser grade of 2 to 5, deformity with moderate scoliosis (Cobb’s angle of 45 to 80 degrees), and a minimum follow-up period of 2 years. Patients with a non-idiopathic etiology (neuromuscular or congenital scoliosis) who were undergoing revision spine surgery were excluded. Among the patients who underwent surgery during the surgery period applicable to each group, all subjects who satisfied the inclusion criteria were randomly included in this study. Medical records for 46 AIS patients who underwent scoliosis correction surgery were included, and these patients were divided into two groups: a monoaxial group (*n* = 23) (Figure 1) and a polyaxial group (*n* = 23) (Figure 2).

### 2.2. Surgical Procedures

All included patients underwent deformity correction by the posterior approach using RD after PSI. We determined the fusion levels according to Suk classification, which divides AIS curves (single thoracic, double thoracic, double major, and thoracolumbar/lumbar curve) into four types. The distal fusion level was determined by the neutral vertebra and end vertebra [1]. The monoaxial or polyaxial pedicle screws were inserted segmentally on both sides of the thoracic and lumbar curves. After a rod was contoured to one-third exaggeration of the normal sagittal alignment, it was inserted into the correction side, and RD was performed by 90° derotation to transform the scoliotic curve to TK or LL. For RD procedures using polyaxial pedicle screw placement, the derotation maneuver was performed on the rotation of the pedicle screw head as a combined rod-and-screw head maneuver. The contoured rod was locked in situ without forceful manipulation on the supportive side after locking the concave rod into the corrective position. Most of the patients in this study underwent thoracoplasty by resecting 2 to 3 inches of each of two to six ribs from the convex side of the apical region of the thoracic curve. The thoracoplasty was performed in AIS patients who had a hump angle of more than 10° and a rip hump height difference of more than 3 cm. Posterior fusion with resected autogenous bone and an allogenuous bone graft were then performed.

### 2.3. Outcome Measures

All patient data were collected from the hospital database. Baseline characteristics included age, body mass index, Risser stage, curve type by Lenke classification, and preoperative radiologic findings. Surgical outcomes were measured including the number of fused segments, thoracoplasty, and rib resection level. Coronal parameters and sagittal parameters of radiological variables included Cobb’s angle of the main curve and the compensatory curve, coronal balance (CB), sagittal vertical axis (SVA), TK, and LL, which were captured by plain radiographs. Rotational parameters of radiological variables included vertebral rotation of the apical vertebra (AV) in the thoracic and lumbar curves, which were measured by a method described by Ho et al. using the axial view from computed tomography (CT) [16]. Clinical outcomes were assessed using Scoliosis Research Society (SRS)-22 questionnaire results from the last follow-up. Complications included chest-tube insertion, hemothorax, pneumonia, infection, wound destruction, abdominal pain, and neurological deficit.

### 2.4. Statistical Analysis

Statistical analysis was performed using SPSS Statistics for Windows, version 21.0 (IBM Corp., Armonk, NY, USA). A normal distribution was confirmed by a Kolmogorov–Smirnov test. After determining data homogeneity or heteroscedasticity, the Mann–Whitney U test was used for continuous variables, and Fisher’s exact test was used for categorical variables, as appropriate. When variables had negative or positive values based on a measured reference point such as CB and SVA, statistical comparisons between groups required converting negative numbers to positive numbers to statistically analyze differences from a reference point. Statistical significance was set at *p* < 0.05.

## 3. Results

### 3.1. Baseline Characteristics and Intraoperative Outcomes

The mean ages were 14.0 years in the monoaxial group and 14.8 years in the polyaxial group with no statistical differences (*p* = 0.115). The preoperative Cobb’s angle of the main curve was 61.2° in the monoaxial group and 60.9° in the polyaxial group (*p* = 0.886). The mean flexibility of the scoliotic curve was 28.9% in the monoaxial group and 31.0% in the polyaxial group with no statistical differences (*p* = 0.442). Baseline characteristics in this study are presented in Table 1. For surgical outcomes, the mean number of fused segments was 12.1 in the monoaxial group and 11.3 in the polyaxial group with no statistical differences (*p* = 0.074). Most patients underwent thoracoplasty with mean rib resection levels of 3.7 in the monoaxial group and 4.2 in the polyaxial group (Table 1).

### 3.2. Radiological Outcomes

The correction rate of Cobb’s angle in the main curve was 70.2% in the monoaxial group and 65.3% in the polyaxial group with statistical significance (*p* = 0.040). Despite statistical differences in the correction rate, both groups showed sufficient correction rates. No statistically significant differences in correction loss were seen between the two groups (*p* = 0.571). Meanwhile, the preoperative Cobb’s angle of the compensatory curve was 36.7° in the monoaxial group and 33.0° in the polyaxial group with no statistical differences (*p* = 0.349). After the deformity correction, the postoperative Cobb’s angle of the compensatory curve was 12.3° in the monoaxial group and 13.5° in the polyaxial group with no statistical differences (*p* = 0.672). For the correction rate of the compensatory curve, no statistical differences were observed between the two groups (*p* = 0.287). The CB was shown within the normal range (−2 cm to 2 cm) with no statistically significant differences between the two groups (all *p* values > 0.05). The SVA also fell within the normal range (−4 cm to 4 cm) with no statistically significant differences between the two groups (all *p* values > 0.05). TK values, which were within a normal range of 20° to 40° for both groups, were significantly higher in the polyaxial group (35.2°) than in the monoaxial group (22.0°), but no statistically significant difference was observed in the correction of TK between the two groups (*p* = 0.939). There were no significant differences in the LL correction between the two groups (*p* = 0.116). For the rotational parameters measured by the axial view in CT, changes in the AV rotation in the thoracic (*p* = 0.865) and lumbar curves (*p* = 0.328) showed no statistically significant differences between the two groups (Table 2).

### 3.3. Clinical Outcomes and Complications

For clinical outcomes at the three-month postoperative follow-up, total SRS-22 scores were 4.2 in the monoaxial group and 4.3 in the polyaxial group with no statistical differences (*p* = 0.531). All scores from the SRS-22 showed no statistically significant differences between the two groups (*p* > 0.05) (Table 3).

With respect to complications, the patients in the monoaxial and polyaxial groups experienced 56.5% and 39.1% chest-tube insertion rates, respectively, with no statistically significant differences (*p* = 0.376). Hemothorax was observed in three of twenty-three patients in the polyaxial group. Only one patient in the monoaxial group and two in the polyaxial group experienced an infection (Table 4).

## 4. Discussion

Since Harrington introduced the derotational spinal maneuver in 1962, RD has been considered the main corrective method to restore coronal and sagittal alignment in patients with AIS [17,18]. A PSI in the thoracic spine, as introduced by Suk et al., is recognized as a safe and reliable method in AIS, making PSI using a posterior approach to RD a mainstay for the surgical treatment of AIS [1,2]. The posteromedialization effect of RD after powerful fixation from PSI is based on the transmission of corrective force from the rod to the pedicle screw to the vertebra [1,19]. Monoaxial pedicle screws have traditionally been used to minimize the loss of correctional forces during transmission [11]. However, the use of polyaxial pedicle screws has recently gained popularity due to their versatility and biomechanical benefits [2,10,20]. In AIS, little information is available on radiological and clinical outcomes according to the type of pedicle screw used. We found that the use of polyaxial pedicle screws provides sufficient correction in surgical treatment using RD to treat AIS patients with a moderate degree of curvature. Moreover, the use of polyaxial screws in the deformity correction did not affect the compensatory curves compared to the use of the monoaxial screws in the patients with AIS. We found no differences in clinical outcomes between the use of monoaxial and polyaxial pedicle screws.

Biomechanical studies have found that monoaxial pedicle screws experience greater pedicle strain compared with polyaxial pedicle screws, producing greater stability [7,8,9,10,11]. Although clinical studies comparing monoaxial and polyaxial pedicle screws are relatively scarce because of the superiority of monoaxial pedicle screws, Kuklo et al., who compared monoaxial (15 patients) versus multiaxial thoracic pedicle screws (20 patients) in the correction of Lenke type I AIS, reported no differences in coronal and sagittal correction, supporting our results [8]. However, contrary to our results, they reported that monoaxial screws offer superior derotation and restoration of thoracic symmetry. For sagittal parameters, Lonner et al. reported finding a trend of loss of TK when using monoaxial pedicle screws, which conflicted with the results of our study [9]. Dalal et al. also presented similar findings with respect to sufficient coronal curve correction when using polyaxial screws and greater apical vertebral rotation when using monoaxial screws [10]. The flexibility of the screw head therefore does not affect the coronal and sagittal alignments in AIS patients.

**Figure 3:**
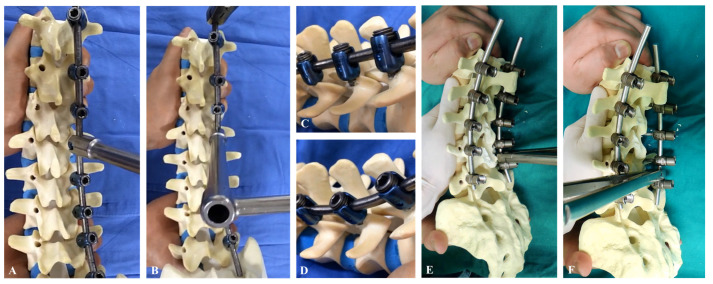
Rod derotation maneuver. (**A**,**B**) Combined rod-and-screw head derotation maneuvers were useful for polyaxial pedicle screws. (**C**,**D**) The self-adaptation property in polyaxial pedicle screws during rod derotation maneuvers. (**E**,**F**) Intraoperative concern of rod derotation, indicating that the derotational force exerted by the rod cannot withstand the purchase monoaxial pedicle screw on the vertebra, which leads to loosening and pull-out of screws by RD procedures.

Our data indicate that the use of polyaxial pedicle screws has a similar effect on coronal, sagittal, and rotational correction compared with the use of monoaxial pedicle screws in moderate scoliotic curvature of AIS. One of the main conflicting results in this study was the superiority of RD in the use of monoaxial pedicle screws for the correction of AIS [8,9,10]. We performed the RD maneuver by applying the screw head and rod, called the combined rod-and-screw head derotation maneuver, which can overcome the biomechanical limitations of polyaxial pedicle screws (Figure 3A,B). This involves the application of derotational force by using the screw-head portion not the rod between the screws. Polyaxial pedicle screws have a self-adaptive property for RD procedures in the correction of AIS (Figure 3C,D). This property may make them a more stable option for correcting AIS, but any advantage should be clarified in future studies. The derotational force exerted by the rod cannot withstand the purchase of a monoaxial pedicle screw on the vertebra, which leads to the loosening and pulling out of screws by RD procedures (Figure 3E,F). RD with monoaxial pedicle screws was based on the correction with volting movement of monoaxial pedicle screws according to the forces in the rod, which can lead to correctional failure. However, RD with polyaxial pedicle screws can reduce the loss of forces because it sequentially acts along with the format of the rod, which makes the RD more flexible. Our technique (combined rod-and-screw head derotation) can therefore serve as an alternative RD maneuver to overcome the biomechanical weakness involved in the surgical correction of moderate scoliotic curves in AIS.

Schlosser et al. reported that the posteromedialization effect of RD procedures can result in residual coronal and axial deformity [21]. With respect to curve correction, derotation may lead to thoracolumbar lordosis [21]. Our data showed no definitive differences in the correction of sagittal and axial parameters. Although the polyaxial pedicle screw theoretically contributed to excluding intraoperative rod-screw engagement, our comparison suggests that the construct of the pedicle screw head did not influence sagittal curve correction [10]. However, the difference in preoperative TK between the two groups weakens our confidence in the results and future studies should address this view of sagittal alignment restoration.

We found no significant differences in clinical outcomes between the use of monoaxial and polyaxial pedicle screws. Long-level fused segments and pedicle screw stiffness may negatively affect a patient’s reported outcome measures in spinal surgery [22,23]. Back pain in particular potentially occurs as a clinical manifestation of pedicle screw-associated complications, which are associated with stiffness of the construct, screw loosening, and non-union [24,25,26,27,28,29]. In contrast, the AIS patients in our study were satisfied with the pain and functions, which may be a function of the characteristics of achieved fusion in pediatrics [2]. Furthermore, the flexibility of the pedicle screw did not affect the clinical outcomes of AIS patients after deformity correction [30].

This study has several limitations. First, our study was performed with a retrospective design and relatively few patients, which can lead to differences in radiological parameters. Second, because we did not include long-term follow-up data, the long-term effect of polyaxial pedicle screws was not determined in this study. Third, although the coronal curve correction rate showed a statistical difference between the two groups, the *p* value was 0.040, close to 0.05. Furthermore, both groups showed a sufficient coronal curve correction rate and no significant difference in the correction rate of the compensatory curve, supporting our conclusions. Therefore, a multi-center, large, comparative, and long-term follow-up study will be required to strengthen our results.

## 5. Conclusions

The use of polyaxial pedicle screws for coronal, sagittal, and rotational correction produced outcomes that were comparable to those produced by monoaxial pedicle screws in surgical treatment using PSI and RD for moderate AIS. Polyaxial pedicle screws are therefore a feasible option for use as a fixation device for the correction of AIS with a moderate degree of curvature.

## Figures and Tables

**Table 1:** Baseline characteristics and operative outcomes between the two groups.

Variables	Monoaxial Group (*n* = 23)	Polyaxial Group (*n* = 23)	*p*
Baseline characteristics			
Gender (Male/Female)	1:22	0:23	1.000
Age (years)	14.0 ± 1.6	14.8 ± 1.8	0.115
BMI (kg/m^2^)	18.6 ± 3.8	19.6 ± 2.9	0.191
Risser stage	3.7 ± 0.5	4.0 ± 0.5	0.129
Lenke type (I:II:III:IV:V:VI)	12:1:7:1:1:1	16:0:4:0:2:1	0.590
Preoperative Cobb’s angle (°)	61.2 ± 13.0	60.9 ± 9.6	0.886
Flexibility (%)	28.9 ± 17.1	31.0 ± 17.8	0.442
Operative outcomes			
Fusion segments (n)	12.1 ± 1.5	11.3 ± 1.4	0.074
Thoracoplasty (Yes/No)	20:3	23:0	0.233
Rib resection level (n)	3.7 ± 1.4	4.2 ± 1.5	0.199

*n*, number.

**Table 2:** Radiological outcomes between the two groups.

Variables	Monoaxial Group (*n* = 23)	Polyaxial Group (*n* = 23)	*p*
*Coronal parameters*			
Cobb’s angle, main curve (°)			
Preoperative (°)	61.2 ± 13.0	60.9 ± 9.6	0.886
Postoperative (°)	17.4 ± 5.4	21.0 ± 5.9	0.043
Correction rate (%)	70.2 ± 5.9	65.3 ± 8.9	0.04
3-month follow-up (°)	17.5 ± 5.1	21.5 ± 4.8	0.046
Correction loss (°)	0.2 ± 0.4	0.5 ± 0.7	0.571
Cobb’s angle, compensatory curve (°)			
Preoperative (°)	36.7 ± 15.4	33.0 ± 10.4	0.349
Postoperative (°)	12.3 ± 9.5	13.5 ± 8.5	0.672
Correction rate (%)	67.0 ± 21.6	60.0 ± 22.4	0.287
Coronal balance (mm)			
Preoperative	15.3 ± 12.3	10.6 ± 7.3	0.344
Postoperative	14.9 ± 12.5	10.3 ± 7.7	0.191
Δ	0.4 ± 14.1	0.3 ± 11.0	0.801
*S* *agittal parameters*			
Sagittal vertical axis (mm)			
Preoperative	32.2 ± 20.4	22.0 ± 20.2	0.072
Postoperative	25.5 ± 15.6	23.7 ± 18.2	0.652
Δ	−6.2 ± 21.0	3.6 ± 28.2	0.144
Thoracic kyphosis (°)			
Preoperative	27.7 ± 11.8	35.2 ± 9.4	0.044
Postoperative	26.2 ± 7.0	33.7 ± 8.3	0.006
Δ	−1.5 ± 11.5	−1.4 ± 8.0	0.939
Lumbar lordosis (°)			
Preoperative	50.0 ± 11.7	44.8 ± 11.2	0.150
Postoperative	48.5 ± 8.8	37.5 ± 9.1	<0.001
Δ	−1.5 ± 11.9	−7.3 ± 9.3	0.116
*Rotational parameters using CT*			
AV in the thoracic curve (°)			
Preoperative	−7.4 ± 7.5	−8.5 ± 4.9	0.546
Postoperative	−8.2 ± 8.1	−9.7 ± 5.1	0.546
Δ	−0.8 ± 7.4	−1.1 ± 7.6	0.865
AV in the lumbar curve (°)			
Preoperative	14.7 ± 11.3	11.9 ± 12.4	0.344
Postoperative	13.7 ± 9.7	8.4 ± 9.2	0.071
Δ	0.8 ± 7.0	4.0 ± 5.1	0.328

*n*, number; CT, computed tomography; AV, apical vertebra.

**Table 3:** Clinical outcomes at the last follow-up between the two groups.

Variables	Monoaxial Group (*n* = 23)	Polyaxial Group (*n* = 23)	*p*
SRS-22, total	4.2 ± 0.4	4.3 ± 0.3	0.531
SRS-22, function	4.6 ± 0.5	4.7 ± 0.4	0.806
SRS-22, pain	4.3 ± 0.6	4.4 ± 0.6	0.554
SRS-22, self-image	4.0 ± 0.5	4.1 ± 0.5	0.756
SRS-22, mental health	3.9 ± 0.6	4.1 ± 0.6	0.374
SRS-22, satisfaction	3.8 ± 0.8	4.1 ± 0.6	0.173

*n*, number; SRS-22, Scoliosis Research Society-22.

**Table 4:** Complications between the two groups.

Variables	Monoaxial Group (*n* = 23)	Polyaxial Group (*n* = 23)	*p*
Chest tube insertion (yes/no)	13:10	9:14	0.376
Hemothorax (yes/no)	0:23	3:20	0.233
Pneumonia (yes/no)	0:23	0:23	1.000
Infection (yes/no)	1:22	2:21	1.000
Wound destruction (yes/no)	0:23	1:22	1.000
Abdominal pain (yes/no)	0:23	0:23	1.000
Neurological deficit (yes/no)	0:23	0:23	1.000

*n*, number.

## Data Availability

Data collected for this study, including individual patient data, will not be made available.
